# Rapid Screening of Cadmium in Rice and Identification of Geographical Origins by Spectral Method

**DOI:** 10.3390/ijerph15020312

**Published:** 2018-02-11

**Authors:** Fang Li, Jihua Wang, Li Xu, Songxue Wang, Minghui Zhou, Jingwei Yin, Anxiang Lu

**Affiliations:** 1 Beijing Research Center for Agricultural Standards and Testing, Beijing Academy of Agriculture and Forestry Sciences, Beijing 100097, China; viki2069@126.com (F.L.); jhwangatfm@163.com (J.W.); llxu159@126.com (L.X.); yinjwfly@126.com (J.Y.); 2 Beijing Municipal Key Laboratory of Agriculture Environment Monitoring, Beijing 100097, China; 3 Academy of State Administration of Grain, Beijing 100037, China; sxxwang@126.com (S.W.); mhmhzhou@126.com (M.Z.)

**Keywords:** X-ray fluorescence, rapid screening, Raman spectroscopy, geographical origin, rice grain

## Abstract

The accuracy, repeatability and detection limits of the energy-dispersive X-ray fluorescence (XRF) spectrometer used in this study were tested to verify its suitability for rapid screening of cadmium in samples. Concentrations of cadmium in rice grain samples were tested by the XRF spectrometer. The results showed that the apparatus had good precision around the national limit value (0.2 mg/kg). Raman spectroscopy has been analyzed in the discrimination of rice grain samples from different geographical origins within China. Scanning time has been discussed in order to obtain better Raman features of rice samples. A total of 31 rice samples were analyzed. After spectral data pre-treatment, principal component analysis (PCA), K-means clustering (KMC), hierarchical clustering (HC) and support vector machine (SVM) were performed to discriminate origins of rice samples. The results showed that the geographical origins of rice could be classified using Raman spectroscopy combined with multivariate analysis.

## 1. Introduction

Rice (*Oryza sativa* L.) is the staple food for more than half the world’s population and serves as the major source of energy [1]. Cadmium is a chronic potent nephrotoxin that is associated with many serious diseases. For non-smokers, large amounts of cereal grain intake is the main way for cadmium to enter the human body, especially for rice grain intake [2,3]. Therefore, it is important to supervise the concentration of cadmium in rice before entering the market for sale or into the grain depot for storage. The traditional method of rice cadmium detection is inductively coupled plasma mass spectrometry (ICP-MS), but its tedious pretreatment, and its time-consuming and specialized operations make it unsuitable for use in grain depot or large wholesale market. A fast, on-site and non-destructive method for detecting the cadmium in rice grains is necessary. Some attempts have been made to use portable near-infrared (NIR) to analyze the trace metal concentration [4,5], but the accuracy was not high enough, because the trace metals have no direct information in the NIR spectrum, their contents need to be derived using other component peaks, which makes it hard to improve testing accuracy. X-ray fluorescence (XRF) spectroscopy has the potential to meet these requirements, owing to its ability to rapidly analyze samples, the simplicity of its sample pre-treatment and operation, high sensitivity, low cost, and its ability to perform in-situ testing [6,7,8]. 

Another main concern in the agricultural/food industry is to ensure the traceability of raw materials and finished products by determining the source [9]. Over the past few decades, fast and non-destructive identification of the geographical origins of different agricultural products have been widely demanded by consumers, producers, and regulatory bodies [10]. NIR spectroscopy has been the most commonly chosen spectroscopic method for the determination of the geographical origins of agricultural products in previous studies [11,12,13,14,15,16,17,18,19,20]. The speed and simplicity of NIR makes it frequently used, although the spectra contain a series of successive overlapping bands that are difficult to assign to particular chemical groups. Raman spectroscopy is an excellent technique for non-destructive analysis, requires little or no sample preparation, and has a very short detection time [21]. It is based on the inelastic scattering between the photons and the sample molecules, causing a frequency shift of an exciting beam of radiation [22]. Raman spectra have good structural selectivity and contain more information needed to elucidate the structure compared to NIR. Raman spectra have fingerprint characteristics, because the energy losses reflect the internal vibrational energies of the scattering molecules, and this property makes them very effective for analysis. Raman bands of rice result mainly from starch and protein vibration, the variability in chemical structure provides valuable spectral features for distinguishing rice samples from different geographical origins. 

Some attempts have been made in the past to characterize and control the quality of rice using scattering spectroscopy [1,23], but no particular attention has been given to regional variability. There is no study focused on the feasibility of on-site rapid screening of cadmium in rice grains by portable X-ray fluorescence (PXRF). There have not been many studies related to geographical distinction of agricultural products with Raman spectroscopy [24,25,26]. Because some of the useful Raman scattering is weak, this makes them buried in the fluorescence produced by the organic compounds in the samples. 

In this work, we verified the accuracy, repeatability and detection limits of the PXRF instrument before testing the cadmium concentration of thirty-one rice samples from different provinces around China. The performance of the instrument can meet the needs of rapid screening. We also evaluate the Raman spectra of the rice samples, the spectra features are analyzed. Chemometric methods have been applied to analyze the spectroscopic information in order to classify the species. K-means clustering (KMC) and hierarchical clustering (HC) are, for the first time, applied to the classification in Raman spectroscopy. The biggest advantage of KMC is its simplicity and speed; and for processing large data sets, the algorithm is relatively scalable and efficient. As for HC, it is not sensitive to the initial data set, and can deal with isolated points and “noise” data.

## 2. Materials and Methods 

### 2.1. Samples Preparation, Apparatus and Measurement Conditions

Samples of dried and polished rice were collected from different cultivating areas in five cities in different provinces around China (Table 1). A total of 31 rice samples were collected, and all the samples were japonica. The standard rice sample GBW(E)100378 (State Administration of Grain, Beijing, China) and a contaminated rice sample (C) were chosen to check the accuracy of the instrument. The standard rice sample GBW(E)100377 (State Administration of Grain, Beijing, China) was selected to verify the apparatus repeatability. The concentration of cadmium in the contaminated sample was detected by inductively coupled plasma mass spectrometry (ICP-MS, Agilent Technologies Inc., Santa Clara, CA, USA). All of the samples were stored for nearly two months before testing. For XRF detection, the samples were ground and sieved with a nylon mesh indoors. A 15.0 g of rice grain sample was packed into a polyethylene cup (D × H: 28 mm × 37 mm, Skyray Instrument Co., Kunshan, China) and covered with 6 µm thick polypropylene film. For Raman detection, the rice grain of each tested sample was transversely cut into thin slices (perpendicularly to the major axis) and positioned on a microscope stage connected to a Raman spectrometer. 

All XRF measurements were made with an energy dispersion PXRF spectrometer (EDX3200SPLUS, Skyray Instrument Co., Kunshan, China) fitted with a W anode X-ray tube, Cu filter and silicon drift detector. The apparatus was operated at a voltage of 66 kV, current of 600 µA and detection of 700 s. Each sample was tested 7 times, and the results were statistically analyzed.

The light scattering detection was operated with Raman spectrometer (DXR Raman microscope, Thermo Fisher Scientific Inc., Waltham, MA, USA) equipped with a 532 nm line of a semiconductor laser with an excitation power of 10 mW at the sample. The spectrometer was equipped with an air-cooled charge-coupled device (CCD) detector with a chip size of 1024 × 768 pixels. The laser was focused on the sample using a microscope setup equipped with a 10×/0.25 objective (Olympus; MPlan, Waltham, MA, USA). Each grain was measured at 5 different spots. A total of 30 grains which were chosen randomly were recorded for each sample. The mean spectrum of 150 spectra was calculated and used for further analysis. 

### 2.2. XRF Detection Performance Verification and Raman Spectral Data Pre-Treatment

The performance of the PXRF spectrometer was validated to ensure its suitability for rapid screening, including accuracy, repeatability and detection limits. The concentrations of cadmium in rice grain samples were tested after verification. In order to increase useful Raman spectral information, mathematical pre-treatment is necessary, including baseline correction and normalization. Background deductions were processed by the testing software: Ominic for dispersive Raman (Thermo Fisher Scientific Inc., Waltham, MA, USA) with a quadratic polynomial. The mean spectrum of each sample was subsequently calculated. The obtained spectra were further normalized using standard normal variate (SNV) [27,28]. SNV is an effective method for correcting additive and multiplicative influence in the spectra. 

### 2.3. Statistical Analysis of Cadmium Concentrations in Rice Grain Samples by XRF

The concentrations of cadmium in all the rice grain samples were tested by XRF. Statistical analysis results include maximum, minimum, average, standard deviation and relative standard deviation. A statistical analysis was also conducted on the cadmium content of five geographical origins, including the items mentioned above as well as concentration range.

### 2.4. Chemometric Methods for Raman Spectra Analysis

Principal component analysis (PCA), K-means clustering (KMC), hierarchical clustering (HC) and support vector machine (SVM) were performed to discriminate the geography of rice samples. All chemometric calculations, including pre-treatment mentioned in Section 2.4. were conducted using MATLAB version 2014a software (The Math-Works Inc., Natick, MA, USA).

#### 2.4.1. PCA

PCA is an analysis method that transforms multiple variables into linear combinations of original variables via linear transformations. The orthogonal transformation is used to transfer the original number of related variables into a set of unrelated new variables [29]. The idea of PCA is to transform the covariance matrix of the original random variable into a diagonal array, and then to reduce the dimensionality of the multidimensional variable. New variables formed by linearly combining the original variables can contain most of the information of the original variable. 

#### 2.4.2. KMC

KMC is a widely used analytical method for finding partitions that minimize the square error measure between the empirical mean of a cluster and the points [30]. All K clusters with the minimized square error guarantee a minimum sum. KMC is an iterative algorithm, the data were considered as points on the K-dimensional space, and clustering analysis is based on distance as a standard. The calculation would not stop until the square error measure drop is not obvious when an iteration is over. This method is applicable to the analysis of large samples, and the number of species should be manually set. 

#### 2.4.3. HC

The HC algorithm combines the two most similar data points in all data points by calculating the similarity between the two types of data points, and iterates the process again and again until all the individuals are classified as one class [31]. It means that the merging algorithm of HC is based on determining the similarity of each class by calculating the distance between each data point and all data points. The smaller the distance, the higher the similarity. The nearest two data points or categories are combined to generate a cluster tree. The key to clustering is the definition of distance between classes. Ward method with Euclidean distance between classes was applied for clustering analysis in this study. 

#### 2.4.4. SVM

SVM is a supervised learning model with associated learning algorithms that analyze data used for classification and regression analysis [32]. It is based on the principle of structural risk minimization. Based on the limited sample information, the best compromise between the complexity of the model information (the learning accuracy of the specific training sample) and the learning ability (the ability to correctly identify any sample) is found to obtain the promotion capability. All the calculations are carried out in the input space with a radial basis function kernel in this research. 

## 3. Results and Discussion

### 3.1. Accuracy, Repeatability and Detection Limits of PXRF Spectrometer 

The standard value of cadmium in GBW(E)100378 is 0.169 ± 0.015 mg/kg, and in the contaminated sample is 2.155 ± 0.202 mg/kg. Both samples were tested 11 times; the results are shown in Table 2. After *t*-test analysis, *t* < *t*_0.05,10_, indicating that there is no significant difference between the detection results of XRF and ICP-MS for the standard sample and contaminated sample.

**Figure 1:**
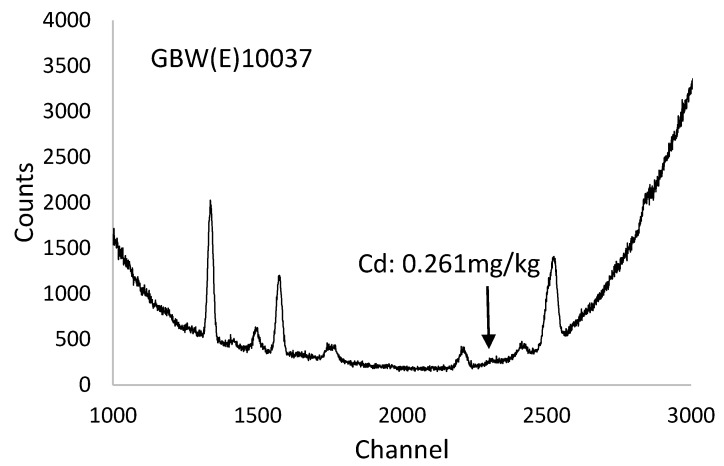
The average XRF spectrum of GBW(E)100377. The spectrum between channels 1000 and 3000 was intercepted to clearly visualize the cadmium peak.

GBW(E)100377 was tested seven times to verify the repeatability of the instrument. The measured results were 0.254, 0.272, 0.296, 0.267, 0.295, 0.268 and 0.307 mg/kg, the calculated arithmetic mean was 0.280 mg/kg, and the concentration range was 0.053. The standard deviation and relative standard deviation were 0.018% and 6.425%, respectively. The average XRF spectrum of GBW(E)100377 is shown in Figure 1. The standard value of cadmium in the sample was 0.261 mg/kg. According to GB/T5009.15-2014, the absolute difference between the results of two independent tests obtained under repeatability conditions should not exceed 20% of the arithmetic mean. That value was 0.280 × 20% = 0.056 in this experiment. In accordance with ISO 5725-6, the critical range is calculated by Equation (1).

(1) $$CR_{0.95}\left(n\right)=f\left(n\right)\sigma_{r}$$

where f(n) is the critical range factor, $\sigma_{r}$ is the sample standard deviation. When *n* is 7, the value of  f(n) is 4.2. $CR_{0.95}\left(7\right)=f\left(7\right)\sigma_{r}=4.2\times 0.0194=0.0815$. So the range (x_max._ − x_min._) < 0.056, range (x_max._ − x_min._) < $CR_{0.95}\left(7\right)$, indicating that the instrument has good repeatability.

To determine the detection limits of the instrument and investigate the reproducibility and accuracy further, a standard rice sample, a contaminated sample and a blank sample were chosen for testing. A confirmatory test was performed with the blank sample to calculate the detection limits. The sample was tested 15 times, and the results are shown in Table 3. The instrument’s qualitative detection limit (QDL) was three times the standard deviation of the blank sample, while the quantitative detection limit (QNDL) was ten times the standard deviation of the blank sample. These results showed that the QDL was 0.0419 mg/kg and the QNDL was 0.1397 mg/kg, which meets the limit standard requirement of 0.2 mg/kg stipulated in the national standard. Therefore, the instrument is able to meet the demands of rapid screening of cadmium in rice.

The performance verification results showed that the PXRF spectrometer used in this study is able to meet the demands of quick screening of cadmium in rice. Once the concentration of cadmium exceeds 0.2 mg/kg, the rice will not be permitted to enter the market or be stored in a grain depot. When the XRF test result is near the limit value, the problematic sample will be sent to the lab to test the chemical concentration and then determine if it is circulated or stored.

### 3.2. Concentration of Cadmium in Rice Grain Samples Detected by XRF

All 31 rice samples were tested by XRF after the performance verification of the apparatus. The detection results are shown in Table 4; samples No. 1–No. 6, No. 7–No. 13, No. 14–No. 18, No. 19–No. 25 and No. 26–No. 31 were from HF, JL, NC, SZ and SZS, respectively. the cadmium concentration in most samples was below 0.2 mg/kg except No. 12 and No. 17, so these two samples should be sent to the lab for testing. No. 18 could also be sent to the lab if necessary. As can be seen from the results, the instrument has good precision above the QNDL, and a small portion of rice had hidden dangers of food safety. Rapid screening of cadmium in rice can identify contaminated or suspicious samples and prevent them from endangering human health.

Cadmium concentration in rice from five different geographical sources was statistically analyzed. The results are shown in Table 5. Origins of the contaminated or suspicious samples were JL and NC. Chemical detection should be carried out to see if there is a need to increase the intensity of the spot check. 

**Figure 2:**
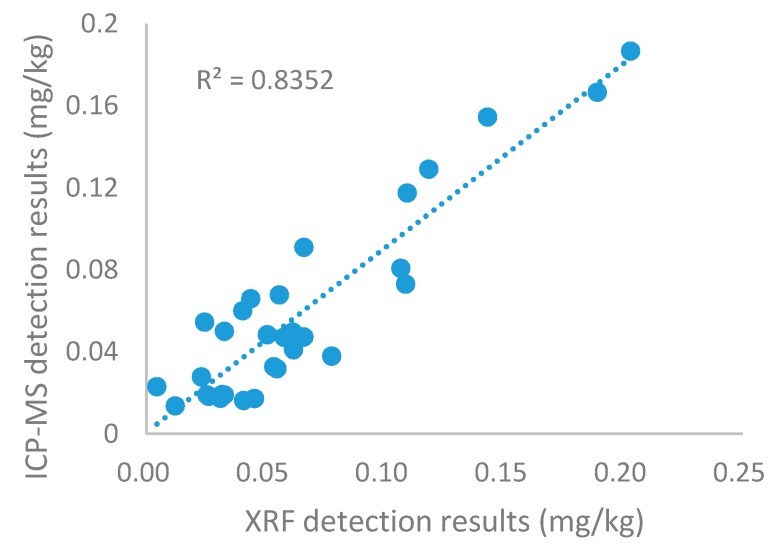
Relationship between XRF detection results and ICP-MS detection results.

The chemical values of cadmium in rice grain samples were tested by ICP-MS, the results were compared to the XRF detection results to calibrate the accuracy and feasibility of this apparatus (Figure 2). The determination coefficient was 0.8352, indicating a high degree of accuracy. The trend of ICP-MS detection results was the same as that of XRF. The concentrations of Nos. 12, 17 and 18 were 0.193, 0.172 and 0.154 mg/kg. The result proves that PXRF analysis is suitable for the rapid screening of cadmium in rice grains. The spectrometer can be used directly in on-site rapid screening to pick out suspicious rice samples to reduce the probability of rice with excessive cadmium content entering the market.

### 3.3. Raman Spectral Analysis

#### 3.3.1. Raman Spectral Pretreatment

**Figure 3:**
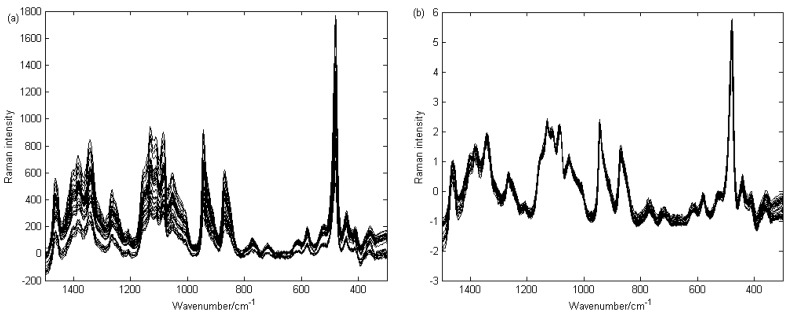
Original (**a**) and normalized (**b**) spectra of 31 rice grain samples. The baseline of the original spectra has been stripped off before normalization.

All spectra were pre-processed, including baseline correction and normalization, before analyzing spectral features or executing further spectral processing. The raw Raman spectra of the 31 samples were shown in Figure 3a. The most noticeable feature is the baseline variation among spectra. As there was no nonlinear background superimposed in the sample spectra, such as fluorescence, the baseline of the spectra were linearly offset to zero at 1500, 1185, 975, 813, 738, 692, 644, 390 cm^−1^, then the baseline-corrected spectra were divided by the corresponding peak area within the range of 1500–300 cm^−1^. The normalization was based on baseline correction using SNV, the normalized spectra that were used for further chemometric methods calculating are shown in Figure 3b.

#### 3.3.2. PCA Analysis for Geographical Origin Discrimination 

**Figure 4:**
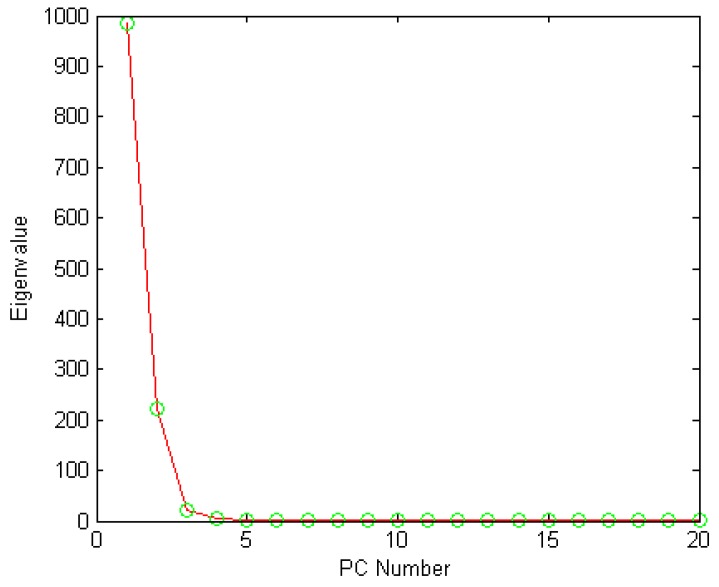
Eigenvalue of all the PCs.

The spectral differences between the samples were analyzed over the 1500–300 cm^−1^ range. The eigenvalue of the PCs was calculated, and the calculation result is shown in Figure 4. The residual variance for each PC occupied the whole variance was computed to confirm the number of PCs used in the calibration model. Each PC got a corresponding score, which would be plotted to check the differences and similarities among each clustering group. Finally, two components were obtained: PC1 accounted for 79.10%, and PC2 accounted for 17.93%.

**Figure 5:**
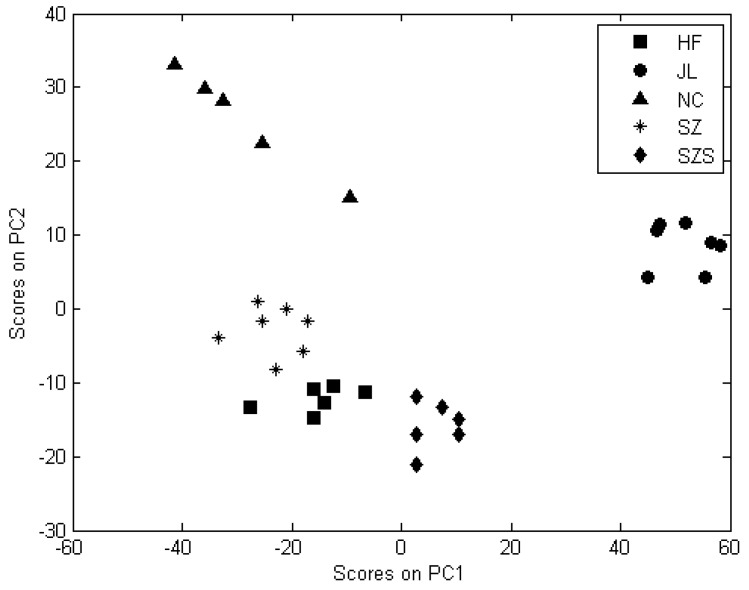
Score scatter plot for the first two PCs of rice grain sample.

The score scatter plot was shown in Figure 5, the species were clearly separated from each other. Rice samples from SZ and HF regions clustered in two groups that were quite close; the reason for this may be that the cultivated areas are adjoining, and that the climate, soil texture and other growing conditions are similar, so that the rice grains have no significant structural and compositional differences, making them very similar to one another.

**Figure 6:**
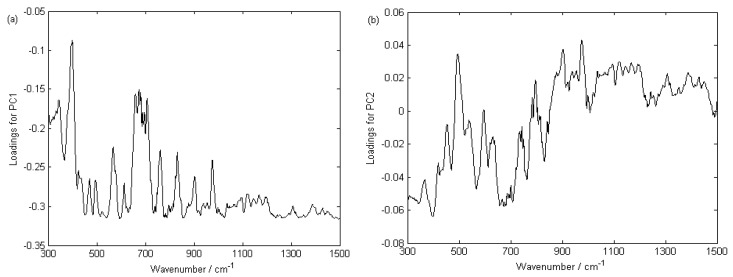
The loading plots for PC1 (**a**) and PC2 (**b**).

Figure 6 shows the loading plot for PC1 and PC2 (1500–300 cm^−1^), which represents the relationship between the PCs and the corresponding original variables. It can be seen from the plot which variables had a greater effect on the difference among the rice grains. The loadings in PC1 all exhibited negative scores, and the highest loadings of PC1 and PC2 had opposite trends compared to Figure 5. The loadings of PC1 changed greatly around 360–365 nm, 445–450 nm, 580–585 nm and 720–725 nm, while PC2 had no obvious change around these bands. The same situation happened to the loadings of PC2 around 390–395 nm, 480–485 nm, 555–560 nm, 875–880 nm and 945–950 nm, with high values, while PC1 did not exhibit much change. This means that PC1 and PC2 can separate the samples efficiently, the conclusion is consistent with the clustering result.

#### 3.3.3. Other Chemometric Methods for Geographical Origin Discrimination

**Figure 7:**
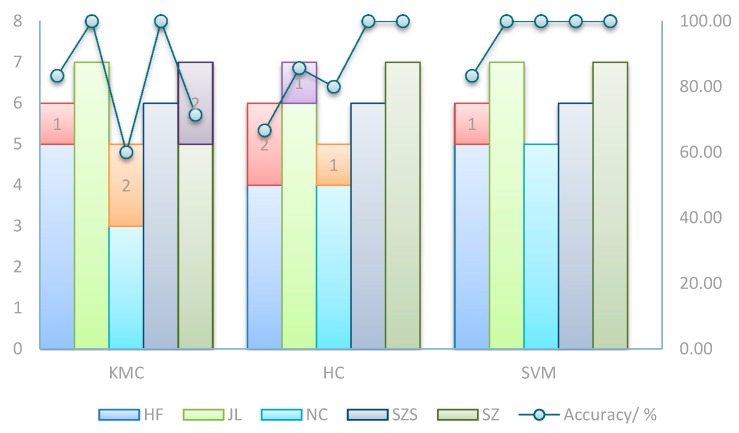
Classification of all grain samples with different chemometric methods. The number marked in the pillar was the misclassification sample number. The polyline shows the classification accuracy of each method for samples in different geographical origin.

KMC, HC and SVM were also performed to classify the samples. Figure 7 shows the classification results and accuracy with the three methods. For rice samples from HF, all the methods had some difficulties with respect to the classification; the accuracy of KMC and SVM was 83.33%, and HC was 66.67%. The other discrimination problems included NC 60% and SZ 71.43% with KMC, and JL 85.71% and NC 80% with HC. For the rest of the samples, all methods had a 100% classification accuracy. Overall, the SVM algorithm shows great superiority over the other two methods.

## 4. Conclusions

The accuracy, repeatability and detection limits of the energy dispersive XRF spectrometer were verified, and the results indicated that the apparatus is able to meet the requirements of quick screening of cadmium concentration in rice grain. Screening can reduce the risk of the problematic rice entering the market or being stored in a grain depot. For Raman analysis, it could be observed from the study that detection time had a significant impact on the Raman features, with proper testing time tending to result in good spectroscopic characteristics. PCA, KMC, HC and SVM were performed based on baseline correction and normalization, with the results showing that rice samples from different geographical origins with these chemometric methods were able to be successfully classified. It is feasible to discriminate the geographical origin using Raman analysis in combination with multivariate methods.

## Figures and Tables

**Table 1:** Geographical information of 31 rice grains samples.

Area/Label		Province	No. of Samples
Hefei	HF	Anhui	6
Jilin	JL	Jilin	7
Nanchang	NC	Jiangxi	5
Shizuishan	SZS	Ningxia	6
Suzhou	SZ	Jiangsu	7

**Table 2:** Accuracy of the instrument. Tested and verified by standard and contaminated sample.

No. of Tests	C Sample (mg/kg)	GBW(E)100378 (mg/kg)
1	2.205	0.184
2	2.612	0.169
3	2.073	0.157
4	2.220	0.170
5	2.212	0.175
6	2.432	0.144
7	2.109	0.176
8	2.210	0.148
9	2.096	0.187
10	2.372	0.172
11	2.011	0.165
Arithmetic mean (mg/kg)	2.232	0.168
Standard value (mg/kg)	2.155	0.169
Relative standard deviation/%	7.932	7.738
*t*	1.442	0.266
*t*_0.05,10_	2.228	

**Table 3:** Instrument detection limits (*n* = 15).

No. of Test	Test Results (mg/kg)	No. of Test	Test Results (mg/kg)
1	0.076	9	0.086
2	0.043	10	0.073
3	0.081	11	0.049
4	0.073	12	0.047
5	0.061	13	0.083
6	0.084	14	0.074
7	0.068	15	0.059
8	0.052		
Arithmetic mean (mg/kg)		0.0673	
Standard deviation		0.014	
Qualitative detection limit (mg/kg)		0.0419	
Quantitative detection limit (mg/kg)		0.1397	

**Table 4:** Detection results of cadmium concentration in rice grain samples by XRF.

Sample Number	Min. (mg/kg)	Max. (mg/kg)	Mean (mg/kg)	SD	RSD/%
1	0.065	0.067	0.066	0.001	1.515
2	0.014	0.040	0.024	0.014	56.697
3	0.031	0.065	0.044	0.029	65.790
4	0.024	0.051	0.040	0.014	35.614
5	0.080	0.132	0.109	0.026	24.301
6	0.083	0.124	0.109	0.023	20.904
7	0.000	0.013	0.004	0.008	173.219
8	0.040	0.077	0.052	0.040	76.923
9	0.036	0.086	0.053	0.028	53.077
10	0.021	0.044	0.033	0.012	35.215
11	0.047	0.069	0.053	0.036	67.924
12	0.199	0.210	0.203	0.006	2.996
13	0.056	0.072	0.065	0.019	29.231
14	0.051	0.063	0.056	0.006	11.549
15	0.109	0.131	0.118	0.011	9.611
16	0.049	0.076	0.061	0.024	39.344
17	0.172	0.205	0.189	0.017	8.742
18	0.158	0.178	0.163	0.016	9.816
19	0.042	0.070	0.055	0.014	25.956
20	0.016	0.088	0.062	0.040	64.383
21	0.052	0.071	0.061	0.016	26.230
22	0.007	0.040	0.023	0.017	71.838
23	0.039	0.093	0.061	0.028	45.952
24	0.001	0.050	0.026	0.025	94.290
25	0.000	0.054	0.033	0.029	87.944
26	0.064	0.068	0.066	0.002	3.030
27	0.096	0.121	0.107	0.013	12.091
28	0.068	0.083	0.078	0.008	10.798
29	0.000	0.039	0.028	0.051	182.142
30	0.012	0.049	0.025	0.021	81.123
31	0.000	0.036	0.012	0.021	173.205

**Table 5:** Cadmium concentration in rice grain samples of different geographical origins by XRF.

Area	Min. (mg/kg)	Max. (mg/kg)	Range (x_max._ − x_min._)	Mean (mg/kg)	SD	RSD/%
HF	0.011	0.132	0.121	0.065	0.038	58.240
JL	0.000	0.210	0.210	0.059	0.066	113.091
NC	0.000	0.205	0.205	0.111	0.059	52.745
SZ	0.000	0.093	0.093	0.041	0.029	69.077
SZS	0.000	0.121	0.121	0.058	0.039	67.284
